# Monkeypox virus: A new etiology for reactive lymphocytes in peripheral blood

**DOI:** 10.1002/jha2.601

**Published:** 2022-10-26

**Authors:** Jared Neeley, Sarah Glogowski, Weina Chen

**Affiliations:** ^1^ Department of Pathology University of Texas Southwestern Medical Center at Dallas Dallas Texas USA

**Keywords:** Monkeypox virus, reactive lymphocytes

1

A 32‐year‐old male with a history of well‐controlled HIV infection (with a normal CD4(+) T‐cell count [501 × 10^6^/L; reference range 495–1635 × 10^6^/L]) presented with acute new‐onset skin lesions (scattered umbilicated papules near mouth and base of bilateral groin area), sore throat, fever, myalgias, and tonsillar/genital ulcerations, with known recent exposure to monkeypox virus. Infectious evaluation demonstrated monkeypox virus (West African clade, by PCR from genital lesion), HSV1 (by PCR) and *Streptococcus dysgalactiae* (from throat sample); SARS‐CoV‐2 and HSV2 were negative by PCR, as was Epstein‐Barr virus by monospot. Review of peripheral blood (PB) smear demonstrated a normal lymphocyte count (3.55 × 10^9^/L) with scattered highly pleomorphic reactive lymphocytes, from immunoblasts with large nuclei, distinct nucleoli, and abundant basophilic cytoplasm (Figure 1A–C) to lymphoplasmacytoid lymphocytes and plasmacytic cells with eccentric nuclei containing condensed chromatin (Figure 1D–F). The patient was treated with tecovirimat, with a subsequent improvement in tonsillar/genital ulcers and decrease in reactive lymphocytes in PB.

**FIGURE 1:**
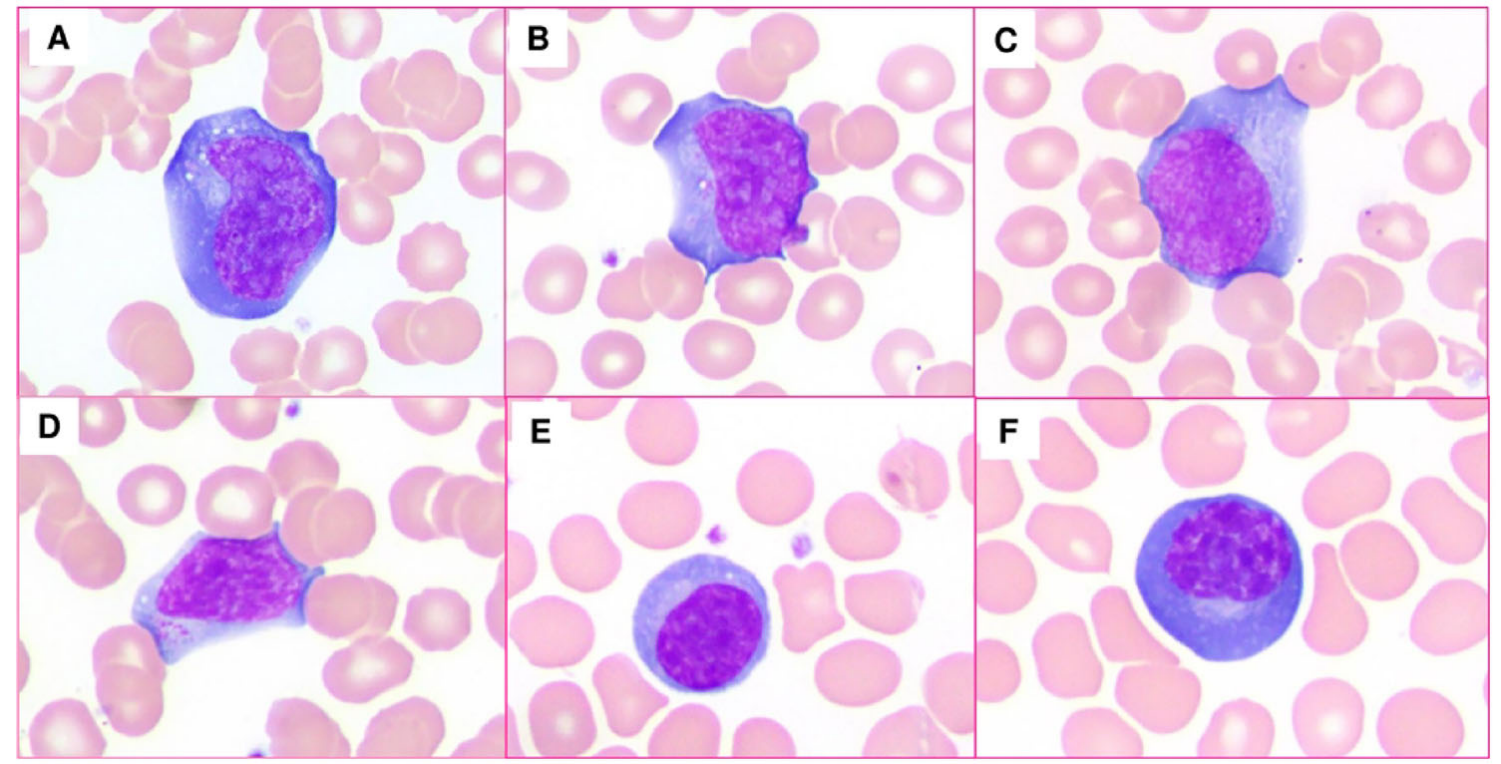
Pleomorphic reactive lymphocytes in peripheral blood film smears. (A–C) Scattered immunoblasts with large nuclei, distinct nucleoli, and abundant basophilic cytoplasm. (D–F) Scattered lymphoplasmacytoid lymphocytes and plasmacytic cells with eccentric nuclei containing condensed chromatin (Wright–Giemsa stain, 1000×)

The monkeypox virus belongs to the Poxviridae family, which also includes the molluscum contagiosum and the smallpox; its outbreak has been reported in more than 37 countries since early 2022 [1]. Tecovirimat may be used for monkeypox infections by preventing formation of the viral envelope by inhibiting p37, a highly conserved protein in all orthopoxviruses. Atypical/reactive lymphocytes could be present in various infections, particularly in acute viral infections. Notably, this patient was co‐infected with monkeypox virus, HIV, HSV1, and Streptococcus. Given this patient's acute onset of presentations including typical skin lesions/umbilicated papules following a recent exposure to monkeypox virus [2], well‐controlled HIV infection with a normal CD4(+) T‐cell count, unknown status of initial versus resolving HSV1 infection, bacterial tonsillitis that is less likely to elicit atypical/reactive lymphocytes, and lack of clinical pharyngitis, atypical/reactive lymphocytes in this case were likely associated with acute monkeypox virus infection. To our knowledge, this is the second reported case study of possible monkeypox associated atypical/reactive lymphocytes in PB. Our findings are similar to those reported recently in which patients had no coinfections with HSV1/2 or varicella‐zoster virus [3]. Awareness of this finding would facilitate early diagnosis by confirmatory molecular testing and prompt future studies for pathophysiology of the disease.

## AUTHOR CONTRIBUTIONS

Jared Neeley, Sarah Glogowski, and Weina Chen wrote and revised the manuscript.

## CONFLICT OF INTEREST

The authors declare they have no conflicts of interest.

## FUNDING INFORMATION

The authors received no specific funding for this work.

## ETHICS STATEMENT

This case followed the institutional patients' ethics. No research on human was performed in this study.

## Data Availability

The data that support the findings of this study are available from the corresponding author upon reasonable request.
